# Coordinated reset vibrotactile stimulation shows prolonged improvement in Parkinson's disease

**DOI:** 10.1002/mds.27223

**Published:** 2017-11-18

**Authors:** Judy Syrkin‐Nikolau, Raumin Neuville, Johanna O'Day, Chioma Anidi, Mandy Miller Koop, Talora Martin, Peter A. Tass, Helen Bronte‐Stewart

**Affiliations:** ^1^ Stanford University Department of Neurology and Neurological Sciences Stanford University School of Medicine Stanford California USA; ^2^ Stanford University Department of Neurosurgery Stanford California USA

**Keywords:** Parkinson's disease, vibrotactile stimulation, coordinated reset, noninvasive, neuromodulation

Coordinated reset stimulation delivers brief high‐frequency trains in a patterned sequence and may reset the phases of neuronal subpopulations toward a desynchronized state.^1^, ^2^, ^3^ Studies have shown that peripheral vibrotactile stimulation accesses central sensory networks and produces a characteristic cortical response.^4^ In this study, we investigated the tolerability and efficacy of peripheral vibrotactile coordinated reset stimulation (PVCRS)^5^ in 5 subjects with idiopathic Parkinson's disease (PD).

Four subjects were off therapy (see Supporting Information); 1 subject was on medication during stimulation. The PVCRS pattern was delivered with C‐2 tactors (Engineering Acoustics Inc.; Supporting Information) to both hands, on all fingers (not the thumb), Figure S1A, and consisted of 3 cycles, each containing a randomized sequence of 4 vibratory bursts equally spaced in time and followed by 2 silent cycles off stimulation (“pause”; Fig. S1B).^5^

**Figure 1:**
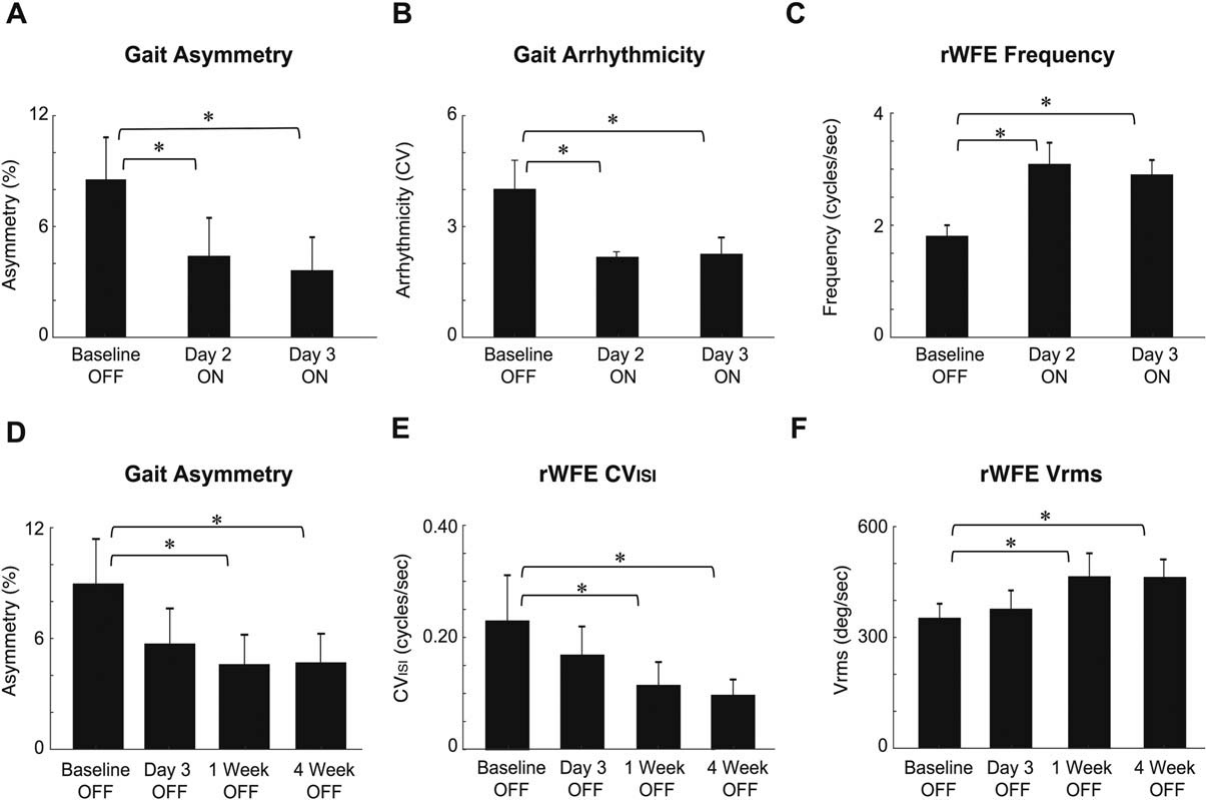
(A‐C) Acute and (D‐F) long‐term effects of peripheral vibrotactile coordinated reset stimulation on gait impairment and wrist bradykinesia. (A) Gait asymmetry and, (B) arrhythmicity decreased significantly from baseline to day 2 and 3 ON stimulation representing more regular gait, while (C) rWFE frequency increased significantly demonstrating diminished wrist bradykinesia. (D) Gait asymmetry decreased significantly while (E) rWFE Vrms and, (F) rWFE CVISI increased significantly from baseline after one week and four weeks indicating the long‐term potency of PVCR. Error bars are standard error of the mean.

The evaluation schedule included off‐therapy testing before stimulation (baseline, day 1), on‐stimulation testing (days 1‐3), and off‐therapy testing (day 3 and 1 and 4 weeks poststimulation; Fig. S1C). Outcomes included a blinded rating of the Unified Parkinson's Disease Rating Scale (motor, UPDRS III), quantitative measures of forward walking using 9‐axis inertial measurement units (APDM Inc.), and the kinematics of repetitive wrist flexion extension (rWFE) using solid‐state gyroscopes (Motus Bioengineering). The acute effect of PVCRS compared outcomes at baseline, off therapy, with those on stimulation, whereas cumulative outcomes compared baseline measures with those off therapy, on day 3, and 1 and 4 weeks after PVCRS.

This study demonstrated that 3 days of PVCRS was safe, tolerable, and resulted in acute and cumulative improvements in quantitative measures of gait impairment and bradykinesia in PD. Gait asymmetry, arrhythmicity, and rWFE frequency improved acutely on stimulation on the second day of stimulation (*P* < 0.001, *P* < 0.001, and *P* = 0.006, respectively) and third day of stimulation (*P* < 0.001, *P* < 0.001, and *P* = 0.016, respectively) compared with baseline (Fig. 1A‐C). There was a cumulative effect of PVCRS on both gait impairment and wrist bradykinesia (Fig 1D‐F). Off therapy, gait asymmetry, wrist rhythm (rWFE CV_ISI_), and angular velocity (rWFE Vrms) were still better than at baseline, 1 week after PVCRS (*P* = 0.001, *P* < 0.05, and *P* = 0.004, respectively) and 4 weeks after PVCRS (*P* < 0.001, *P* < 0.05, and *P* = 0.006, respectively). One subject, who was on medication during stimulation, also demonstrated long‐term improvement in gait asymmetry and arrhythmicity. No significant effect was found on the blinded UPDRS III scores across the group.

To our knowledge this is the first demonstration that PVCRS is tolerable and efficacious in PD. There was acute (on stimulation) and cumulative (off therapy) improvement in gait and bradykinesia in PD. The cumulative benefit suggests that peripheral CR stimulation may have a persistent desynchronizing effect on sensorimotor networks, as demonstrated using subthalamic electrical CR neurostimulation.^6^

The improvement in 1 subject, stimulated on medication, suggests that a future PVCRS trial may be possible on medication (Table S2). A sham stimulation condition will be important in future trials to minimize the placebo effect, although this was less likely to have contributed to the cumulative improvement.

## Author Roles

Judy Syrkin‐Nikolau: research project conception, organization, and execution; statistical analyses design and execution; preparation of manuscript.

Raumin Neuville: research project execution.

Johanna O'Day: statistical analysis, design, and execution.

Chioma Anidi: statistical execution.

Mandy Miller Koop: statistical analysis, design, and execution.

Talora Martin: research project execution.

Peter Tass: corresponding author; development of vibrotactile CR stimulation concept and device; research project conception; preparation of manuscript.

Helen Brontë‐Stewart: corresponding author; research project conception, organization and execution; statistical analyses design and execution; preparation of manuscript.

## Financial Disclosures

Judy Syrkin‐Nikolau — funding: John A. Blume Foundation, Robert and Ruth Halperin Foundation.

Raumin Neuville — none.

Johanna O'Day — funding: Stanford Bio‐X Bowes Graduate Student Fellowship.

Chioma Anidi — funding: NINDS R21 NS096398‐02.

Mandy Miller Koop — funding: Michael J. Fox Foundation; consultant for Cleveland Clinic.

Talora Martin — funding: NIAAA R01 AA023165‐04.

Peter Tass — funding: John A. Blume Foundation, donation by Pelham Olive, Helmholtz Society, donations by Jim and Connie Binns; consultant for Boston Scientific in the field of DBS for movement disorders and SCS for pain management; main inventor of a patent portfolio for invasive and noninvasive CR stimulation owned by Juelich Research Center, Germany and Stanford University. Helen Brontë‐Stewart — funding: John E. Cahill Family Foundation, Robert and Ruth Halperin Foundation, John A. Blume Foundation, Helen M. Cahill Award for Research in Parkinson's Disease, Michael J. Fox Foundation, NIAAA R01 AA023165‐04, the NINDS R21 NS096398‐02.

## Supporting information

Additional Supporting Information may be found in the online version of this article at the publisher's website.

Supporting Information

Supporting Information

Supporting Information

Supporting Information
