# Supplementary figures and images for: Coordinated reset vibrotactile stimulation shows prolonged improvement in Parkinson's disease
Source: Mov Disord. 2017 Nov 18;33(1):179–80. doi: 10.1002/mds.27223 (PMC5836884; doi:10.1002/mds.27223)

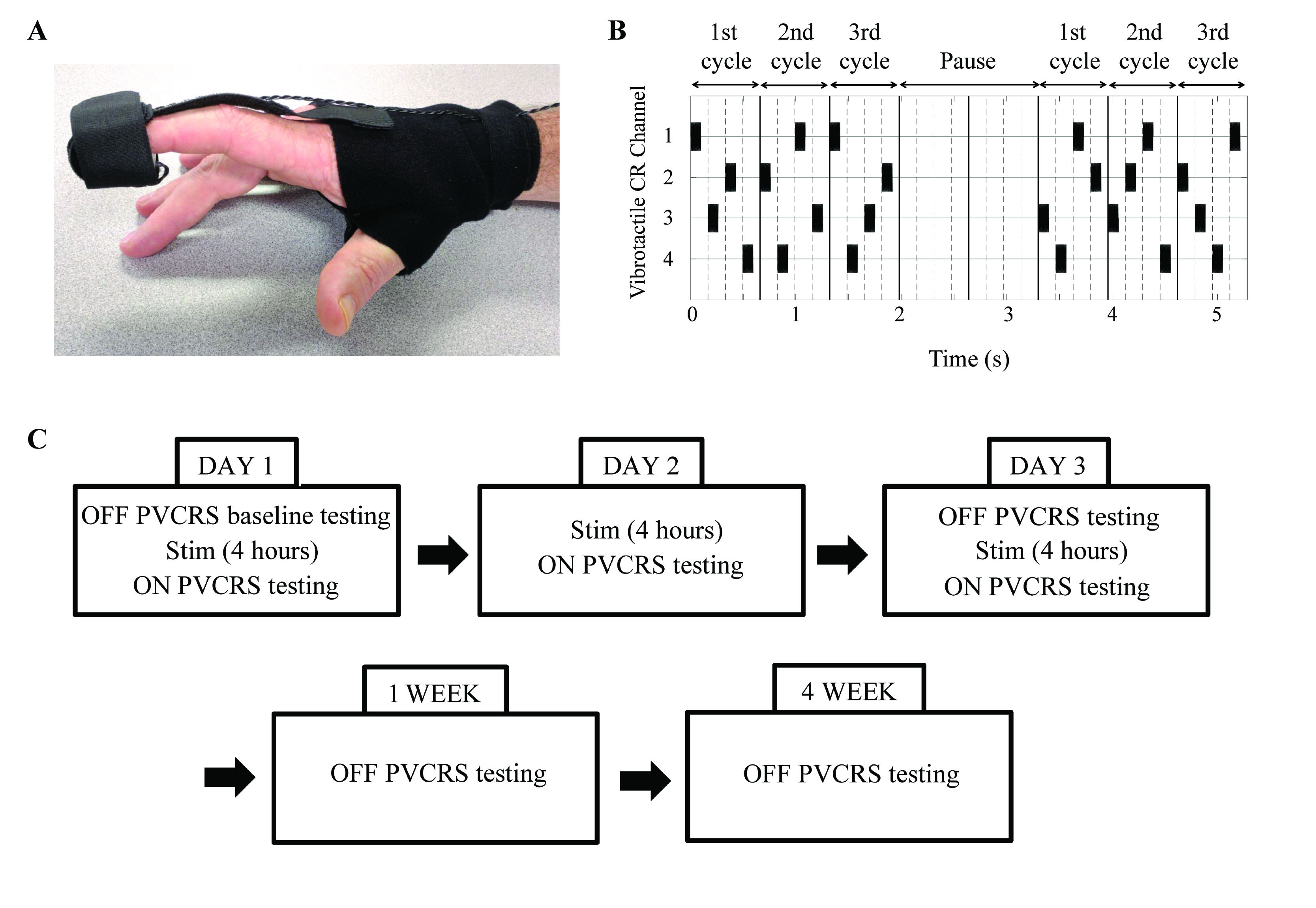

Supplement: Supplementary file 1 — Supporting Information [file MDS-33-179-s001.tif]
