# Supplementary material for: Coordinated reset vibrotactile stimulation shows prolonged improvement in Parkinson's disease
Source: Mov Disord. 2017 Nov 18;33(1):179–80. doi: 10.1002/mds.27223 (PMC5836884; doi:10.1002/mds.27223)
Supplement: Supplementary file 2 — Supporting Information [file MDS-33-179-s002.docx]

Table S1: Demographic data for the five subjects

| **Pt** | **Phenotype** | **Age** | **Disease Duration (years)** | **Baseline UPDRS III OFF Therapy and Stimulation** |
| --- | --- | --- | --- | --- |
| 1 | TD | 58 | 2.25 | 21 |
| 2 | AR | 61 | 8.92 | 3 |
| 3 | TD | 69 | 3.92 | 28 |
| 4 | TD | 67 | 7.58 | 37 |
| 5 | TD | 75 | 5.92 | 16 |

Table S1. Blinded UPDRS III scores do not include rigidity or speech. TD = tremor dominant, AR = akinetic rigid.
