# Supplementary material for: Coordinated reset vibrotactile stimulation shows prolonged improvement in Parkinson's disease
Source: Mov Disord. 2017 Nov 18;33(1):179–80. doi: 10.1002/mds.27223 (PMC5836884; doi:10.1002/mds.27223)
Supplement: Supplementary file 3 — Supporting Information [file MDS-33-179-s003.docx]

Table S2: Gait Asymmetry and Arrhythmicity for the subject stimulated on medication

| **Visit medication/stimulation** | **Asymmetry** | **Arrhythmicity** |
| --- | --- | --- |
| **Day 1 off/OFF** | 4.30 | 2.51 |
| **Day 1 on/OFF** | 2.79 | 1.64 |
| **Day 2 on/ON** | 2.81 | 2.08 |
| **Day 3 on/OFF** | 3.98 | 1.99 |
| **Day 3 on/ON** | 1.26 | 1.51 |
| **1 Week off/OFF** | 3.25 | 2.64 |
| **4 Week off/OFF** | 3.37 | 1.25 |
