# Supplementary material for: Coordinated reset vibrotactile stimulation shows prolonged improvement in Parkinson's disease
Source: Mov Disord. 2017 Nov 18;33(1):179–80. doi: 10.1002/mds.27223 (PMC5836884; doi:10.1002/mds.27223)
Supplement: Supplementary file 4 — Supporting Information [file MDS-33-179-s004.docx]

**Supporting Material**

**Human Subjects**

All subjects had idiopathic Parkinson’s disease (PD) and consented to participate in a trial of peripheral vibrotactile coordinated reset stimulation (PVCRS). Subjects were excluded from the trial if they had significant cognitive decline or other comorbidities that interfered with their movement. The subjects’ mean age was 66 +/- 6.8 years, and were Hoehn Yahr Stage II or III, Table S1.

[Insert Table S1]

The primary outcome was safety and tolerability of undergoing three days of four hour periods of PVCRS. The secondary outcome variables were the UPDRS III score, gait arrhythmicity and asymmetry, and the rWFE metrics. The efficacy of PVCRS was assessed by comparing the baseline off therapy UPDRS III and kinematics to those ON stimulation. The long-term effect of PVCRS was assessed by comparing the off therapy UPDRS III and kinematics at baseline, before the third day of stimulation, and at one and four weeks after stimulation.

**Experimental Protocol**

Subjects received PVCRS on the fingertips for four hours on days 1, 2, and 3, totaling twelve hours over all three days. Due to hardware constraints, vibratory bursts were delivered to four different fingers (all fingers except for the thumb) of both hands with C-2 tactors (EAI Engineering Acoustics Inc., Casselberry, FL). The C-2 tactors were fixed with Velcro tape (Figure S1A), and the constant indentation of the stimulator’s contactor surface was 0.5 mm. The PVCRS pattern consisted of three cycles, each containing a randomized sequence of four vibratory bursts, equally spaced in time and followed by two silent cycles off stimulation (“pause”, Figure S1B). The vibratory bursts had a vibration frequency of 250 Hz and vibration amplitude of 0.35 mm. The vibration amplitude was linearly ramped up within 40 s after PVCRS onset. PVCRS cycle duration was 660 ms, whereas vibratory burst duration was 100 ms. The 3 cycles on, 2 cycles off pattern was repeated periodically^1,2,3^. The random variation of the vibratory burst sequences^2^ and the 3:2 ON-OFF pattern^1,3^ were used to enhance the desynchronizing PVCRS effect. The PVCRS pattern was delivered to both hands, so that the same fingers of both hands were stimulated at the same time.

Subjects had a total of five study visits throughout the duration of the trial, which consisted of visits on three consecutive days, as well as at one and four weeks post-stimulation. Figure S1C details the evaluation schedule, which consisted of off therapy baseline testing before stimulation was started (day 1), ON stimulation testing (days 1-3), and off therapy testing (day 3, one and four weeks post stimulation). Four subjects were off medication (24 hours for long-acting and 12 hours for short acting dopaminergic medication) during the stimulation and at all evaluations. Three of these four subjects did not take any medications in between days 1, 2, and 3 by choice, and the fourth subject had deep brain stimulation (DBS) and turned his DBS back on overnight on days 1, 2, and 3. This subject was off DBS therapy for at least 15 minutes prior to starting OFF PVCR baseline testing. We have previously demonstrated that neural activity returns to baseline within 14 seconds and most motor signs return to baseline within several minutes after turning off DBS^4,5^. If there were any residual therapeutic effects of high frequency DBS, it would bias our results toward increased performance in the baseline movement, underestimating the true benefit of PVCR. The fifth subject was on medication at all visits except the baseline off therapy and one and four week follow-ups. Time points on medication for this subject were excluded from analysis.

[Insert Figure S1]

**Figure S1.** System design and implementation. A) C-2 tactor glove B) Peripheral Vibrotactile CR stimulation (PVCRS) pattern C) Subject visit schedule.

Assessment of the effect of PVCRS included the Unified Parkinson’s disease Rating Scale motor assessment (UPDRS III, minus rigidity and speech), assessed by a blinded rater, quantitative measures of forward walking (FW) and repetitive wrist flexion extension (rWFE), using wearable sensors. The self-paced rWFE task consisted of thirty seconds of repetitive flexion and extension; the subjects were instructed to flex and extend their hands at the wrist as quickly as possible after a “Go” command and to stop only when instructed. For the FW task, subjects walked forwards for 10m, turned around, returned, and repeated this for a total of 40m of straight walking. Subjects were also given a customized adverse effects (AEs) questionnaire which asked about any AEs they were experiencing before, during, and after the stimulation at each visit.

**Data Acquisition and Analysis**

Angular velocity during rWFE was measured using wearable gyroscopic sensors attached to the dorsum of each hand (Motus Bioengineering, Inc., Benicia, CA) and monitored by continuous video^6^. The rWFE angular velocity data was sampled at 1000 Hz and video was recorded at 30 frames/second. Root mean square velocity (Vrms), frequency (cycles/second), coefficient of variation (standard deviation divided by the mean) of Vrms (CV_Vrms_), coefficient of variation of the interstrike interval (CV_ISI_), and coefficient of variation of distance (angular range) per cycle (CV_dist_) were calculated for each movement epoch.

Kinematic data during FW was recorded using six 9-axis wireless Opal® inertial measurement unit (IMU) sensors (APDM, Inc., Portland, OR, USA), attached to the top of each foot, to each shank, and to the lumbar, and chest trunk regions. Sampling rate for the IMU sensors was 128 Hz. The gait measures were calculated using the gyroscope (angular velocity) signals from the shank IMUs. Care was taken to align the sensor on the shank, so that the positive Z axis was approximately lateral and recorded gait angular velocity in the sagittal plane. The data were filtered using a zero phase 8^th^ order low pass Butterworth filter with a 9Hz cut-off frequency and a principal components analysis was used to align the shank angular velocity with the sagittal plane. Using the aligned Z angular velocity, the beginning of the swing phase (positive slope zero crossing), end of swing phase (subsequent negative slope zero crossing) were identified. Swing and stride times were calculated from these time points. Swing and stride times were then used to calculate arrhythmicity and asymmetry. Arrhythmicity and asymmetry were calculated using periods of straight walking. Asymmetry and arrhythmicity were defined as: asymmetry = 100 × |ln(SSWT/LSWT)|, where SSWT and LSWT correspond to the leg with the shortest and longest mean swing time over the trials, respectively and arrhythmicity = the mean stride time coefficient of variation (CV) of both legs^7,8^. A large stride time CV is indicative of a less rhythmic gait. Analysis was performed in LabVIEW (National Instruments, Inc.) and MATLAB (The MathWorks, Inc.). The UPDRS III was scored by a blinded rater, with rigidity excluded. The subject was not blinded to the stimulation, as they could easily feel when the stimulation was on or off.

To limit the number of comparisons among time points for the OFF to ON, immediate stimulation effect, we focused on comparing baseline testing OFF stimulation with the second and third day ON stimulation testing points (after a total of 8 and 12 hours of vibrotactile CR stimulation, respectively). Similarly, for the long-term effect analysis, we focused on comparing baseline OFF testing to the day 3 OFF, 1 Week OFF, and 4 Week OFF time points. The statistical analysis of the immediate and long-term effect of stimulation in the OFF therapy state excluded the subject who was on medication during the trial. A one-way repeated measure ANOVA or one-way repeated measure ANOVA on Ranks and Dunnett’s Method was used for both analyses. Turns during the FW task were removed from analysis, which resulted in 4 straight walking FW time segments. In order to account for this, a linear mixed effects model was used. For the immediate effect, visit day was a fixed effect and a factor variable with 3 levels (Baseline OFF, day 2 ON, and day 3 ON). For the long-term effect, visit day was also a fixed effect, and a factor variable with 4 levels (Baseline OFF, day 3 OFF, 1 Week OFF, and 4 Week OFF). For both linear mixed effects models, subject number was a random effect, and a random intercept was used in the model. Residuals were assessed for homoscedasticity and normality, and all statistical assumptions were met.

**Supporting Results**

One subject performed the evaluations off therapy but was unable to stay off medication while on peripheral vibrotactile stimulation (PVCRS). The ON stimulation data was excluded from the group analysis. Table S2 details the gait data for this subject. Gait arrhythmicity and asymmetry improved on compared to off medication on day 1, no PVCRS. There appeared to be improvement in asymmetry, on medication/ON PVCRS (on/ON), on day 3 compared to the on/OFF on day 1 and on/ON on day 2. The on medication kinematic metrics of the rWFE task remained relatively constant ON and OFF PVCRS throughout days 1, 2, and 3, data not shown. The subject did show long-term improvement in both rWFE and FW metrics off/OFF at the one and four week visits that followed the same trend in long-term improvement as the rest of the group, when PVCRS was performed in the off medication state.

[Insert Table S2]

References

1. Tass P. A model of desynchronizing deep brain stimulation with a demand-controlled coordinated reset of neural subpopulations. Biol Cybern 2003;89:81–88.
2. Tass P, Majtanik M. Long-term anti-kindling effects of desynchronizing brain stimulation: a theoretical study. Biol Cybern 2006;94:58–66.
3. Lysyansky B, Popovych O, Tass P. Desynchronizing anti-resonance effect of m: n ON-OFF coordinated reset stimulation. J Neural Eng 2011;8:036019.
4. Trager M et al. Subthalamic beta oscillations are attenuated after withdrawal of chronic high frequency neurostimulation in Parkinson's disease. Neurobiol Dis 2016;96:22-30.
5. Temperli P, Ghika J, Villemure JG, Burkhard PR, Bogousslavsky J, Vingerhoets FJ. How do parkinsonian signs return after discontinuation of subthalamic DBS?. Neurology 2003;60(1):78-81.
6. Koop M, Andrzejewski A, Hill B, Heit G, Bronte-Stewart H. Improvement in a quantitative measure of bradykinesia after microelectrode recording in patients with Parkinson’s disease during deep brain stimulation surgery. Mov Disord Off J Mov Disord Soc 2006;21:673–678.
7. Plotnik M, Giladi N, Balash Y, Peretz C, Hausdorff J. Is freezing of gait in Parkinson’s disease related to asymmetric motor function? Ann Neurol 2005;57:656–663.
8. Plotnik M, Giladi N, Hausdorff J. A new measure for quantifying the bilateral coordination of human gait: effects of aging and Parkinson’s disease. Exp Brain Res 2007;181:561–570.
